# Evening and morning alterations in Obstructive Sleep Apnea red blood cell proteome

**DOI:** 10.1016/j.dib.2017.01.005

**Published:** 2017-01-16

**Authors:** Amélia Feliciano, Fátima Vaz, Cristina Valentim-Coelho, Vukosava M. Torres, Rita Silva, Vesna Prosinecki, Bruno M. Alexandre, Andreia Almeida, Catarina Almeida-Marques, Ana S. Carvalho, Rune Matthiesen, Atul Malhotra, Paula Pinto, Cristina Bárbara, Deborah Penque

**Affiliations:** a Serviço de Pneumologia, Centro Hospitalar Lisboa Norte (CHLN), Lisboa, Portugal; b Laboratório de Proteómica, Departamento de Genética Humana, Instituto Nacional de Saúde Dr Ricardo Jorge, Lisboa 1640-016, Portugal; c ToxOmics- Centre of Toxicogenomics and Human Health, Universidade Nova de Lisboa, Portugal; d Departamento da Promoção da Saúde, Instituto Nacional de Saúde Dr Ricardo Jorge, Lisboa 1640-016, Portugal; e Pulmonary, Critical Care and Sleep Medicine Division, University of California San Diego, CA, USA; f Instituto de Saúde Ambiental (ISAMB), Faculdade de Medicina, Universidade de Lisboa, Portugal

**Keywords:** Obstructive Sleep Apnea, Red blood cells, 2D-DIGE, Biomarkers

## Abstract

This article presents proteomics data referenced in [1] Using proteomics-based evaluation of red blood cells (RBCs), we have identified differentially abundant proteins associated with Obstructive Sleep Apnea Syndrome (OSA). RBCs were collected from peripheral blood of patients with moderate/severe OSA or snoring at pre- (evening) and post-night (morning) polysomnography, so that proteome variations between these time points could be assessed. RBC cytoplasmic fraction depleted of hemoglobin, using Hemovoid^™^ system, were analyzed by two-dimensional fluorescence difference gel electrophoresis (2D-DIGE), the 2D image software-based analyzed and relevant differentially abundant proteins identified by mass spectrometry (MS). MS identified 31 protein spots differentially abundant corresponding to 21 unique proteins possibly due to the existence of post-translational modification regulations. Functional analysis by bioinformatics tools indicated that most proteins are associated with catalytic, oxidoreductase, peroxidase, hydrolase, ATPase and anti-oxidant activity. At morning a larger numbers of differential proteins including response to chemical stimulus, oxidation reduction, regulation of catalytic activity and response to stress were observed in OSA. The data might support further research in OSA biomarker discovery and validation.

**Specifications Table**

**Table:** 

Subject area	Biology
More specific subject area	Molecular Medicine; Clinical Proteomics
Type of data	Figures, graphics and tables
How data was acquired	Two-dimensional fluorescence difference gel electrophoresis (2-D DIGE) - based proteomics followed by image analysis with Progenesis SameSpots, version 4.5 software (Nonlinear Dynamics, UK). Protein identification by mass spectrometry (MALDI/TOF/TOF). Pathway analysis by open source DAVID software [2].
Data format	Filtered, analysed
Experimental factors	RBC samples were hemoglobin (Hb) depleted, using Hemovoid^™^ system, before analysis.
Experimental features	Samples from OSA and Snorers (controls) patients biobank collected at evening and morning time (i.e, before and after night polysomnographic diagnosis) were enrolled.
Data source location	Lisbon, Portugal,
Data accessibility	Data is with this article

**Value of the data**

• For the first time (evening/morning) changes in OSA RBC proteome are shown probably induced by nocturnal intermittent hypoxia and sleep disruption experienced by these patients.
• The provided data set may help to get new insights into RBC homeostasis which dysregulation can be a source of oxidative-stress and/or inflammation causally linking OSA to cancer and cardiometabolic disorders.
• These data could be used in further verification/validation assays to selected candidates biomarker of OSA severity and/or treatment response.

## 1 Data

2-D gel reference image of OSA RBC cytoplasmic fraction depleted from hemoglobin was shown. Graphics representing the identified variations for the different PRDX2 and Catalase proteoforms between groups and conditions were highlighted as examples. The 31 proteins spots identified by MALDI/TOF/TOF MS are displayed in detail in the Table. Fold-change histograms and pathway analysis between patients’ groups and conditions were shown. Material and methods are provided in detail as much as possible to be reproduced elsewhere.

## 2 Experimental design, materials and methods

### 2.1 Sample collection and biochemical analysis

Patients with suspected OSA are hospitalized for an overnight polysomnography (PSG) study. For the proteomics study [1], blood samples were collected into EDTA-coated polypropylene tubes before and after PSG, i.e., between 8:00 pm and 09:30 pm (referred as ‘evening’) and between 7:30 am and 09:00 am (referred as ‘morning’). Samples were kept no longer than 4 h at 4 °C until fractionation by centrifugation. The obtained RBC pellets were aliquoted and stored at −80 °C until analysis.

### 2.2 RBC sample preparation

RBC pellet samples were lysated by incubation with (1:20) 5 mM phosphate buffer pH 7,4 containing 1:100 cocktail of protease inhibitors (P8340, Sigma Aldrich) for 30 min at 4 °C followed sonication 10 s/40 amplitude/pulse mode (Ultrasonic Processor, VibraCell, Sonics & Materials Inc, USA). After centrifugation at 25.000 *g* for 30 min at 4 °C, the supernatant cytoplasm fractions were recovered for further hemoglobin (Hb) depletion using Hemovoid depletion columns (Biotech Support Group, Monmouth JCT, USA), according to the manufacture׳s protocol. The obtained Hb depleted fractions were concentrated and buffer-exchanged with 25 mM NH_4_HCO_3_ pH 8.4 by centrifugal filtration using 3-kD Molecular Weight Cut-Offs (MWCO) (Amicon Ultra 4, Millipore) spin concentrators. The protein concentration was determined by a colorimetric assay (Pierce BCA Protein Assay Kit, Thermo Fisher) according to the manufacturer׳s protocol. The efficiency of Hb-depletion was confirmed by analysing samples (10 μg/lane) on coomassie stained 4–12% SDS-PAGE mini gels (NuPAGE Novex Bis Tris, Invitrogen, USA) (data not shown). Samples were stored at −80 °C until further analysis.

### 2.3 2D-DIGE

Analysis of Hb-depleted pooled samples (n=3/group) from the four study groups was based on a 2D-DIGE approach, using the CyDye DIGE fluor minimal dyes Cy3 and Cy5 from GE Healthcare. Briefly, 50 μg of each pooled samples (in duplicate) were lyophilized and resuspended in 6.25 ul of lysis buffer (7 M urea, 2 M thiourea, 2% (w/v) 3-[(3-cholamidopropyl) dimethylammonio]- 1-propanesulfonate (CHAPS) and incubated with 400 pmol Cy5 solution for 30 min at 4 °C in the dark. The labelling reaction was stopped by adding 1 μL of 10 mM lysine and the samples were incubated for another 10 min. A sample of each pool was pooled together and labelled with Cy3 as above described to comprise the internal standard (IS) for each gel. Each Cy5 labelled sample was combined with an equal amount of Cy3 labelled IS sample (50 μg:50 μg) and mixed with lysis buffer for with a trace of bromophenol blue to 140 μl final volume. To ensure an optimal focusing of the proteins, an ampholyte solution for pH 3–10 NL (Serva, Heidelberg, Germany) was added in a concentration of 1% and the samples were loaded to 24-cm IPG strips with a pH gradient of 3-10NL (GE Healthcare) previously rehydrated for 20 h at RT with 310 μl of lysis buffer. IEF was performed in an Ettan IPGphor 3 (GE Healthcare) in ceramic manifold with cup loading of the sample, and focused as follows: step and hold 100 V for 5 h, gradient 300 V for 2 h, gradient 500 V for 1 h, gradient 1000 V for 2 h, gradient 2000 V for 1 h, gradient 6000 V for 3 h and 8000 V for 2 h, followed by step and hold at 8000 V for 8 h. The maximum current per strip was set to 50 μA. Prior to 2nd dimension stripes were equilibrated once with 8 mL of SDS equilibration buffer (6 M urea, 75 mM Tris–HCl pH 8.8, 29.3% glycerol (87%), 2% SDS and a trace of bromphenol blue) including 1% of DTT (15 min, RT) in order to accomplish reduction of disulfide bonds, followed by derivatization of cysteine residues with equilibration buffer containing 4% of iodacetamide (15 min, RT).

Second-dimension separation was performed using Ettan DALT six electrophoresis system (GE Healthcare) using 12.5% SDS-PAGE running overnight with a power of 1 W per gel and at a constant temperature of 15 °C. A preparative 2D-gel stained with coomassie blue, containing equal amount of each non-labeled pooled samples mixed with 50 μg of IS labeled sample, in a total of 700 μg of proteins, was performed for further spot cutoff for mass spectrometry (MS) protein identification. Introducing some labeled sample into preparative gel facilitates gel match with analytical gels for spot location and picking for MS analysis.

### 2.4 2D-DIGE image analysis

Each gel was scanned at 100 μm resolution using an Amersham Biosciences Typhoon 8400 variable imager, resulting in two images, one for the IS and one for the sample. To improve the signal collection capabilities of the instrument and avoid image saturation, a prescan was performed to check and adjust the photomultiplier tube (PMT) voltages of the different channels that were set to values between 500 and 550 V.

**Fig. 1:**
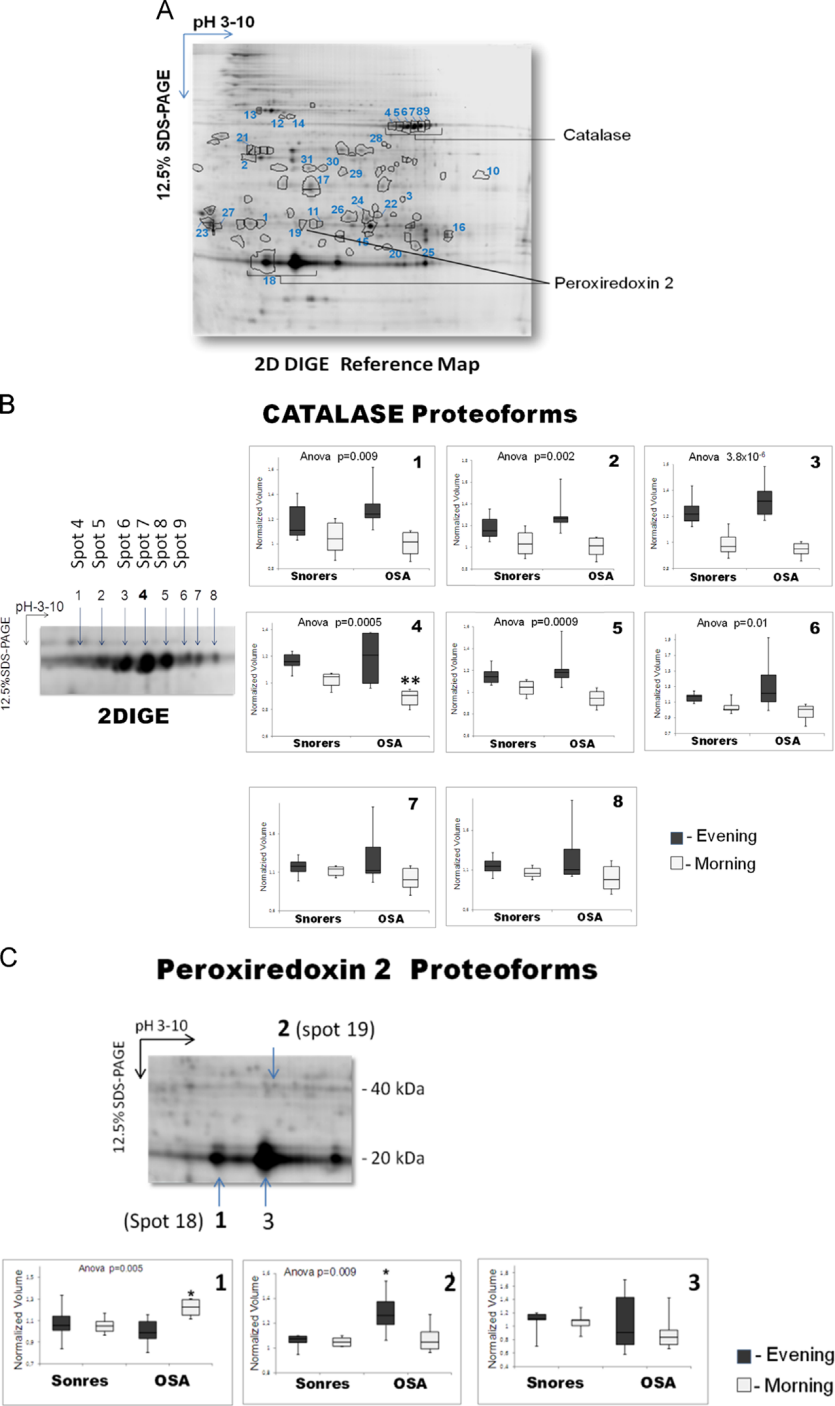
2DIGE reference map of Hb-depleted RBC from OSA and Snorers patients collected at evening or morning day time. (A) The 31 protein spots exhibiting significant differences in abundance (fold change ≥ 1.2; Anova *p*<0.05) between OSA and Snorers at evening or morning times that were identified by MS, corresponding to 21 unique proteins in consequence of post-translational modification (PTM) regulations are indicated with circles on the 2D gel image. The identity of these proteins is fully described in the Table 1. As an example, catalase proteoforms **(B)** and peroxiredoxin-2 proteoforms **(C)** are described in more detail. **(B)** About 8 proteoforms for catalase were identified. The most acidic ones (spot 4–9 or n°1–6) probably resulting from phosphorylation [4] were shown significantly decreased in OSA morning samples. **(C)** At least three peroxiredoxin-2 protoforms were identified. The acidic monomeric forms (spot 18 or n° 1) and dimeric forms (spot 19 or n°2) reported as oxi/overoxidized forms of peroxiredoxin-2 [5] were signficantly increased in OSA RBC at morning evening daytime, respectively.

Spot detection, gel matching, and statistical analysis were performed with Progenesis SameSpots, version 4.5 (Nonlinear Dynamics, UK). Abundance values of matched spots across all gel images, expressed as normalized volume, were compared between groups, so that each spot could be assigned a score of relative significant difference, in terms of *p* value (<0.05). Relative content alteration of each spot across the study groups was expressed by fold change values, which were calculated by the ratio of the mean normalized volumes of a certain spot in each condition. Spots decreasing their abundance were represented by negative fold values, calculated as the inverse of the previous ratio multiplied by −1 (Fig. 1).

### 2.5 Protein identification

After in-gel digestion of protein spots collected from the preparative gel, proteins were identified by MS. The protein spot picking was performed manually. In-gel digestion was performed as described before [2]. Tryptic peptides, prepared in 50% (v/v) ACN and 0.1% (v/v) trifluoroacetic acid (TFA), were directly deposited on a 192-well matrix-assisted laser desorption/ionization (MALDI) plate with 5 mg/ml α-cyano-4-hydroxycinnamic acid (α- CHCA, 1:1), prepared in 0.1% TFA/60% ACN (v/v) and allowed to co-crystallize at RT. Peptides were analyzed on an Applied Biosystems 4700 MALDI Proteomics Analyzer (with time-of-flight/time-of-flight (TOF/TOF) ion optics exactly as described before [2], [3]. The identified proteins are displayed in the Table.

### 2.6 Protein annotation and classification

**Fig. 2:**
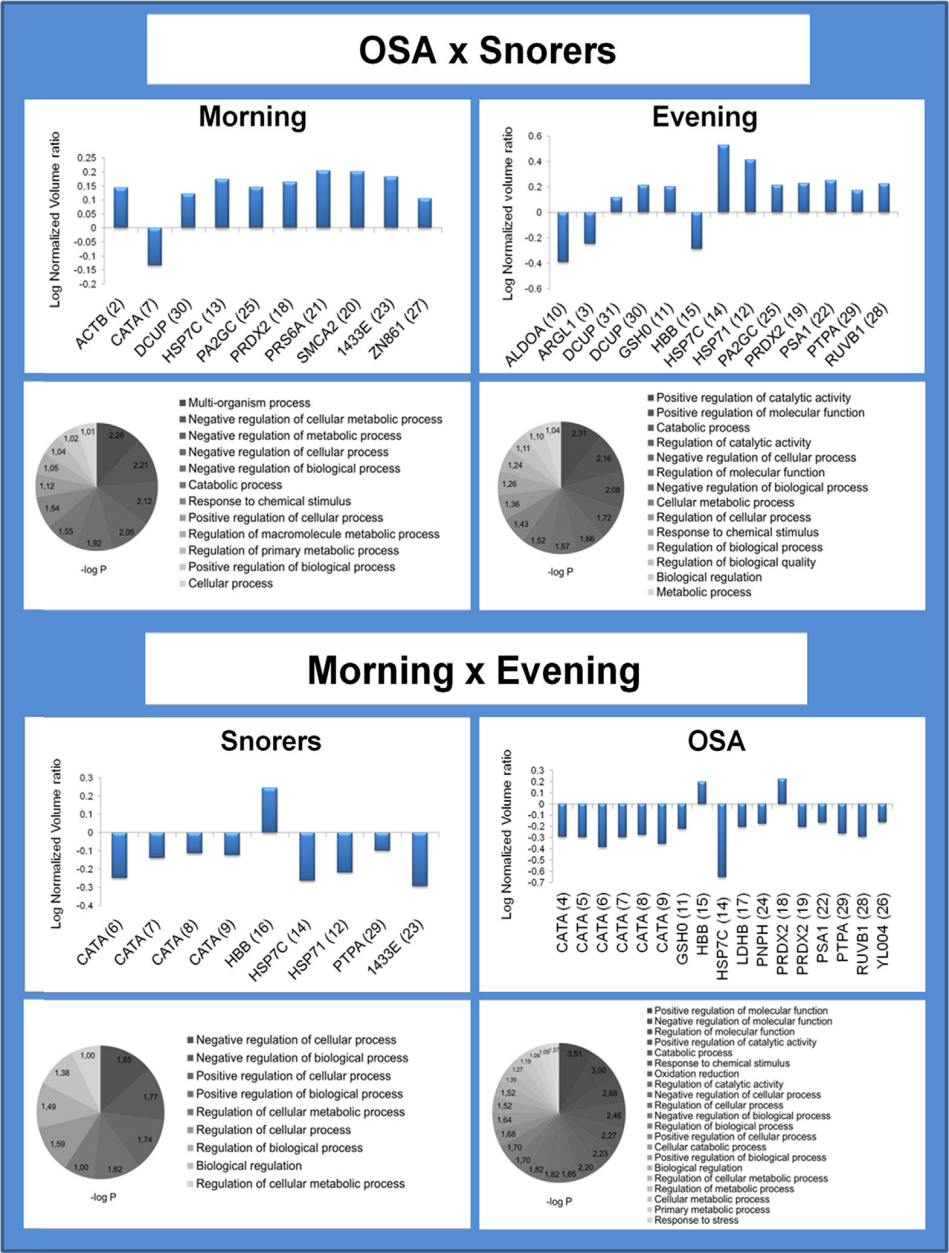
Fold-change histogram and pathway analysis of RBC proteins/proteoforms with differential abundance in OSA *versus* Snorers at morning or evening and Morning *versus* Evening in Snorers or OSA (read from top to down). Proteins/proteoforms with significant differential abundance from each comparable group/condition were plotted on the histograms, which the log volume ratio of (±) 1.0 represents a 10-fold increase/decrease changes. The most representative biological processes retrieved from DAVID v6.7 [6] are shown on the bottom of each respective histograms.

Protein annotation properties were acquired using the Database for Annotation, Visualization and Integrated Discovery (DAVID) v6.7 [6]. This open-source tool retrieves a set of biological and functional information such as GO terms, subcellular location, molecular function and association with biological process and/or disease with p-values of over-representation ≤0.05 (Fig. 2) 

## Figures and Tables

**Table 1:** Annotation of differential RBC cytosolic proteins between OSA and Snorers at Evening and Morning.

**Spot**	**Protein name**	**Acession ID**	**MW/pI**	**Mascot Score**	**Sequence Coverage (%)**	**Anova (p)**	**Fold (Anova *p*<0.05)**
1	**Actin**	**ACTB_HUMAN**	42052/5.29	73	20	0.016	1.4
2	**Actin**	**ACTB_HUMAN**	42052/5.29	26	2	0.042	1.2
3	**Arginine and glutamate-rich protein 1**	**ARGL1_HUMAN**	33197/10.35	60	11	0.036	1.5
4	**Catalase**	**CATA_HUMAN**	59719/6.90	663	25	0.009	1.3
5	**Catalase**	**CATA_HUMAN**	59719/6.90	253	7	0.002	1.3
6	**Catalase**	**CATA_HUMAN**	59719/6.90	896	34	3.8E-06	1.4
7	**Catalase**	**CATA_HUMAN**	59719/6.90	730	31	4.9E-04	1.3
8	**Catalase**	**CATA_HUMAN**	59719/6.90	797	29	0.001	1.3
9	**Catalase**	**CATA_HUMAN**	59719/6.90	397	21	0.012	1.4
10	**Fructose-bisphosphate aldolase A**	**ALDOA_HUMAN**	39851/8.30	76	24	0.034	1.8
11	**Glutamate--cysteine ligase regulatory subunit**	**GSH0_HUMAN**	30708/5.69	235	26	0.020	1.2
12	**Heat shock 70 kDa protein 1A/1B**	**HSP71_HUMAN**	70294/5.48	201	29	0.007	1.8
13	**Heat shock cognate 71 kDa protein**	**HSP7C_HUMAN**	70854/5.37	52	2	0.019	1.2
14	**Heat shock cognate 71 kDa protein**	**HSP7C_HUMAN**	70854/5.37	380	23	3.8E-04	2.1
15	**Hemoglobin subunit beta**	**HBB_HUMAN**	16102/6.75	142	63	0.005	1.3
16	**Hemoglobin subunit beta**	**HBB_HUMAN**	16102/6.75	61	8	0.024	1.4
17	L-lactate dehydrogenase B chain	**LDHB_HUMAN**	36615/5.72	412	32	0.003	1.3
18	**Peroxiredoxin-2**	**PRDX2_HUMAN**	22049/5.66	857	64	0.031	1.2
19	**Peroxiredoxin-2**	**PRDX2_HUMAN**	22049/5.66	85	29	0.004	1.2
20	**Probable global transcription activator SNF2L2**	**SMCA2_HUMAN**	181166/6.76	34	4	0.019	1.3
21	**26 S protease regulatory subunit 6 A**	**PRS6A_HUMAN**	49458/5.13	117	30	0.006	1.4
22	**Proteasome subunit alpha type-1**	**PSA1_HUMAN**	29822/6.15	166	39	0.001	1.2
23	**14-3-3 protein epsilon**	**1433E_HUMAN**	29155/4.63	96	4	0.007	1.4
24	**Purine nucleoside phosphorylase**	**PNPH_HUMAN**	32097/6.45	542	40	0.019	1.2
25	**Putative inactive group IIC secretory phospholipase A2**	**PA2GC_HUMAN**	16833/8.84	13	14	0.016	1.2
26	**Putative uncharacterized protein FLJ45999**	**YL004_HUMAN**	18957/10.14	32	10	0.012	1.2
27	**Putative zinc finger protein 861**	**ZN861_HUMAN**	11989/8.91	30	17	0.037	1.2
28	**RuvB-like 1**	**RUVB1_HUMAN**	50538/6.02	64	19	4.7E-04	1.3
29	**Serine/threonine-protein phosphatase 2 A activator**	**PTPA_HUMAN**	40641/5.63	246	17	1.1E-04	1.3
30	**Uroporphyrinogen decarboxylase**	**DCUP_HUMAN**	40761/5.77	32	2	0.007	1.3
31	**Uroporphyrinogen decarboxylase**	**DCUP_HUMAN**	40761/5.77	134	8	0.010	1.2
